# Kinetically-Controlled Growth of Chestnut-Like Au Nanocrystals with High-Density Tips and Their High SERS Performances on Organochlorine Pesticides

**DOI:** 10.3390/nano8070560

**Published:** 2018-07-23

**Authors:** Xia Zhou, Qian Zhao, Guangqiang Liu, Hongwen Zhang, Yue Li, Weiping Cai

**Affiliations:** 1 Key Lab of Materials Physics, Anhui Key Lab of Nanomaterials and Nanotechnology, Institute of Solid State Physics, Chinese Academy of Sciences, Hefei 230031, China; zhouxia@issp.ac.cn (X.Z.); zhaoqian142336@yeah.net (Q.Z.); hwzhang@issp.ac.cn (H.Z.); yueli@issp.ac.cn (Y.L.); 2 Science Island Branch of Graduate School, University of Science and Technology of China, Hefei 230026, China; 3 School of Chemistry and Chemical Engineering, Suzhou University, Suzhou 234000, China

**Keywords:** kinetically-controlled seed growth, chestnut-like Au nanocrystals, high-density tips, SERS performances, chlorinated pesticide residues

## Abstract

A modified seed growth route was developed to fabricate the Au nanocrystals with high-density tips based on kinetically-controlled growth via adjusting the adding rate of Au seeds into growth solution. The obtained Au nanostructures were chestnut-like in morphology and about 100 nm in size. They were built of the radial [111]-oriented nanoneedles and were 30–50 nm in length. There were about 120–150 tips in each nanocrystal. The formation of chestnut-like Au nanocrystals is ascribed to surfactant-induced preferential growth of seeds along direction [111]. Importantly, the chestnut-like Au configuration displayed powerful surface enhanced Raman scattering (SERS) performance (enhance factor > 10^7^), owing to the high density of tips. Further, such film was used as a SERS substrate for the detection of lindane (γ-666) molecules (the typical organochlorine pesticide). The detection limit was about 10 ppb, and the relationship between SERS intensity *I* and concentration *C* of 666 accords with the double logarithm linear. This work presents a simple approach to Au nanocrystal with high-density tips, and provides a highly efficacious SERS-substrate for quantitative and trace recognition of toxic chlorinated pesticides.

## 1. Introduction

Surface enhanced Raman scattering (SERS)-based detection is a powerful and ultrasensitive technique, and possesses extensive applications in areas such as molecular imaging [1], biomedical science [2,3,4], environmental monitoring [5,6], food safety [7,8], and biological sensing [9,10,11], etc. One of the key issues for SERS-based detection is the fabrication of the substrates with high SERS activity or enhancement factor (EF) and stable structure. Most of them are noble metals with nanostructure. The sub-nanometer sharp tips on the metal substrates are the most important enhancement structure [12,13,14]. Such tips serve as the nano antennas which are not only the “super electromagnetic intensifiers” or the “hot spots”, but also the preferential adsorption sites for the molecules, and amplify the Raman signal [15,16]. Therefore, the preparation of the noble metal substrates with the high number density of the tips is an important way to obtain high SERS activity. 

Au or Ag SERS substrates with the well-defined tipped “hot-spots” have been reported mainly via chemical reduction and seed-assisted growth methods [16,17,18,19,20,21,22,23,24,25,26]. Specifically, Wang et al. [16] fabricated Au nanostars via a seed-assisted growth approach, and demonstrated the powerful SERS performance ascribed to nano-engineering “hot spots” on their tipped surface. Fang et al. [17] reported the fabrication of the sea urchin-like Au microparticle arrays via the reduction of HAuCl_4_ by Fe suspension solution, and showed strong SERS activity with one or two orders of magnitude over that of the individual microparticle. Also, they obtained aggregated hollow Au-Ag alloy nanourchins with about 100 nm in size using L-dopa as the reductant. Such alloy nanourchins with about 70–100 tips for each one, showed a significant enhancement of the electromagnetic field [18]. Generally, for the chemical reduction method, a proportion of the precursors need to be adjusted accurately, and the morphologic uniformity and controllability are relatively limited. The seed-assisted growth method is simple and easy to operate and can be used for synthesis of nanostars with few tips, but it is difficult to obtain the high number density of tips, such as urchin or flower-like nanocrystals.

Herein, we present a modified seed growth approach to fabricate Au configuration with high-density tips derived from kinetically-controlled growth via adjusting the addition rate of seeds into growth solution. The obtained Au nanostructure is chestnut-like in shape and about 100 nm in size. Such chestnut-like nanocrystals consist of the radial nanoneedles with [111] orientation and mostly about 30–50 nm in length. There are about 120–150 tips in each nanocrystal. We found that adding conditions of seed solution, including adding rate and amount, are crucial to the formation of the chestnut-like Au nanocrystals. Importantly, such chestnut-like Au nanocrystal-built film with high-density tips has displayed powerful SERS performance (EF > 10^7^). When it is used as a SERS substrate for the detection of lindane (γ-666) molecules (typical organochlorine pesticide), a detection limit of about 10 ppb is achieved. Moreover, the relationship between SERS intensity *I* and concentration *C* of γ-666 accords with the double logarithm linear. This work presents a simple approach to the Au nanostructure with high-density tips, and provides a highly efficacious SERS-substrate for the quantitative and trace recognition of toxic chlorinated pesticides.

## 2. Materials and Methods

### 2.1. Materials

Gold(III) chloride (HAuCl_4_·4H_2_O), sodium borohydride (NaBH_4_), silver nitrate (AgNO_3_), Cetyl trimethyl ammonium chloride (CTAC), 4-aminothiophenol (C_6_H_7_NS, 4-ATP), Lindane, L-ascorbic acid (AA), hydrochloric acid, and ethanol were analytical reagents and purchased from Aladdin Corporation. Ultrapure water (18.2 MΩ resistivity) was provided by Millipore Milli-Q equipment.

### 2.2. Fabrication of Chestnut-Like Au Nanocrystals

Au seeds were firstly prepared by reducing HAuCl_4_ with NaBH_4_ in the presence of CTAC, as previously reported [16]. Briefly, 0.30 mL ice-cold, freshly prepared 10 mM NaBH_4_ aqueous solution was injected into 10 mL aqueous solution with CTAC (0.10 M) and HAuCl_4_ (0.25 mM) at 20 °C. The solution was gently stirred for 2 min in order to make the solution homogeneous, and then stopped, un-disturbed for 2 h. The seed-containing solution was diluted to 1000-fold with 0.1 M CTAC aqueous solution, and used as the seed solution for subsequent preparation of the chestnut-like Au nanocrystals.

The growth solution was then prepared by adding 0.5 mL HAuCl_4_ (10 mM), 0.1 mL AgNO_3_ (10 mM), 0.2 mL HCl (1.0 M), and 0.5 mL AA (0.02 M) into the CTAC (10.0 mL, 0.1M) aqueous solution. Here, the small amount of Ag^+^ ions was introduced only for decreasing the growth rate, as previously reported [16]. Fifty μL diluted Au seed solution was subsequently injected into the growth solution using a micro-injection pump at 25 μL/min with stirring. Then the solution was undisturbed for 4 h at 20 °C, and the color of the solution changed from colorless to purple. The obtained products were washed and dispersed with water.

### 2.3. Characterization

The optical absorbance spectrum was measured with a Shimadzu UV-2600 spectrometer. The morphology and microstructure of the products were observed on a field emission scanning electron microscope (FESEM, FEI Sirion 200) and transmission electron microscope (TEM, JEM-2100). X-ray diffraction (XRD) patterns were recorded on a diffractometer (X’Pert Philips) with Cu Kα radiation (0.15406 nm).

## 3. Results and Discussion

**Figure 1:**
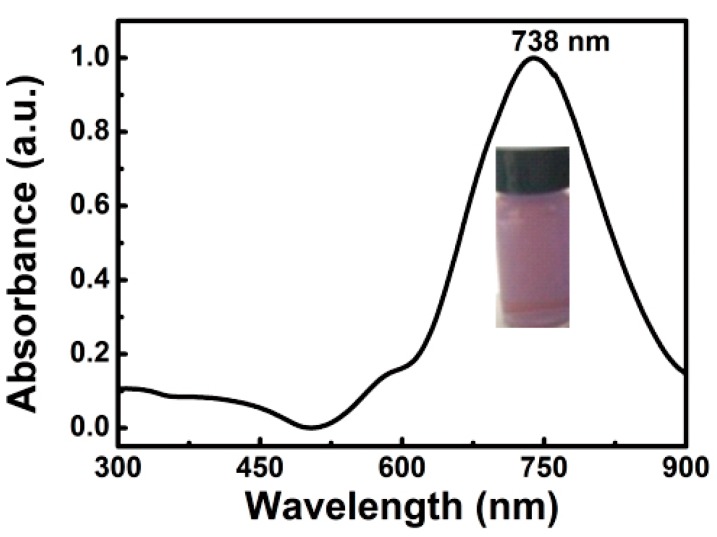
The optical absorbance spectrum and the photo (inset) of the Au nanocrystal colloidal solution.

After the diluted Au seeds were injected into the growth solution for 4 h, a purple solution was obtained, as illustrated in the inset of Figure 1. The corresponding optical absorbance spectrum is shown in Figure 1. In addition to a small shoulder around 570 nm, a strong main peak is located at 738 nm, which could originate from the hybridization of localized surface plasmon resonance (SPR) of Au nanoparticles [17].

### 3.1. Morphology and Structure

**Figure 2:**
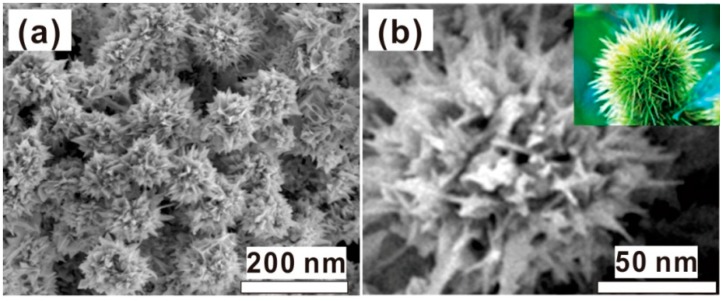
The field emission scanning electron microscope (FESEM) images of the as-prepared products. (**a**) The image with low magnification. (**b**) The magnified image of the single particle in (**a**). The inset is the photo of one chestnut for reference.

The absolute yield of the products was estimated to be about 90% by weighing and mole number in the growth solution. The morphology of the products is shown in Figure 2. The products consist of the chestnut-like particles with about 100 nm in size (Figure 2a). Such chestnut-like particles are composed of the radial nanoneedles about 30–50 nm in length, as more clearly illustrated in Figure 2b. For individual chestnut-like particles, there were about 120–150 tips or nanoneedles, in addition to the many sharp protrusions and nanogaps. Due to the chestnut-like morphology, the small shoulder peak around 570 nm in Figure 1 should originate from the hybridization of plasmons localized at the cores of the Au nanoparticles, while the main peak at 738 nm results from the increasing aspect ratios of the branches or radial nanoneedles, according to previous reports [16,17,18].

**Figure 3:**
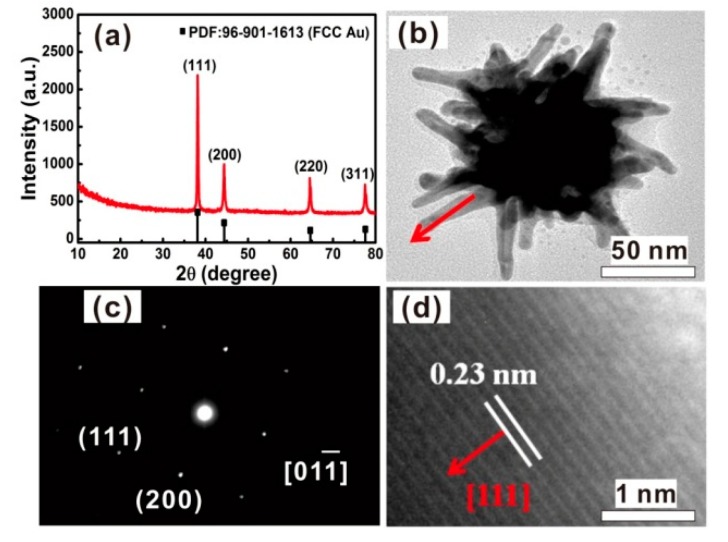
(**a**) XRD pattern of the chestnut-like Au nanocrystals. The line spectrum is the standard pattern of Au powders. (**b**) TEM image of an individual chestnut-like Au nanocrystal. (**c**) Selected area electron diffraction (SAED) pattern of the particle in (**b**). (**d**) HRTEM image of the nanoneedle marked with an arrow in (**b**). The arrows in (**b**,**d**) are parallel.

Correspondingly, the XRD analysis was conducted for the products, as shown in Figure 3a. Four characteristic diffraction peaks were observed at 38.2°, 44.4°, 64.6°, and 77.5°, which respectively correspond to (111), (200), (220), and (311) planes of face-centered cubic (FCC) Au (JCPDS, No. 96-901-1613). Further, the microstructure was examined. Figure 3b shows the typical TEM image of an individual chestnut-like particle. The needles were about 50 nm in length and <10 nm in width. The selected area electron diffraction (SAED) revealed that the chestnut-like particles were single crystalline, as demonstrated in Figure 3c. Figure 3d shows the high-resolution TEM (HRTEM) image corresponding to one nanoneedle marked in Figure 3b, and demonstrated that the nanoneedle is [111]-oriented.

**Figure 4:**
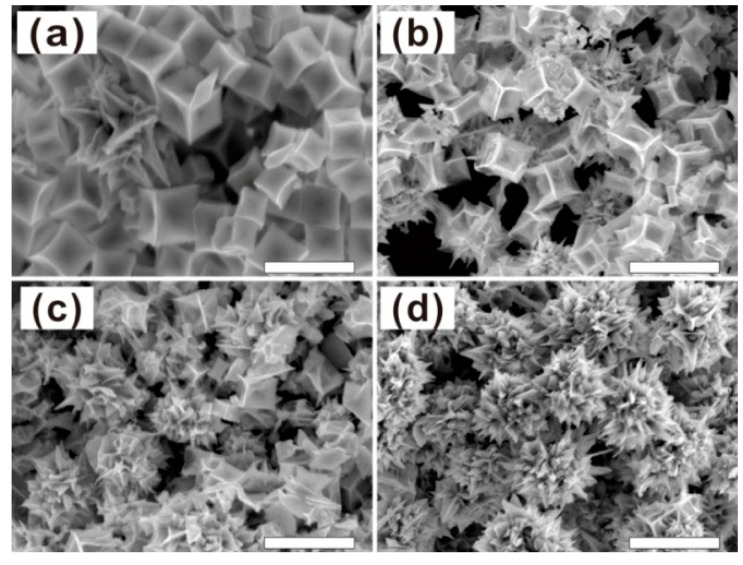
FESEM images of the products obtained after reaction for different durations. (**a**) 1 h, (**b**) 2 h, (**c**) 3 h, (**d**) 6 h. The scale bars are 200 nm.

In order to reveal the formation process of chestnut-like Au nanocrystals, the morphological evolution with the reaction time was studied. Figure 4 shows the morphologies of the products after the Au seeds were injected into the growth solution for different durations. When the reaction time was 1 h or less, the products were concave nanocubes (Figure 4a). With increasing the reaction time to 2 h, the nanocubes become more concaved, accompanied with appearance of the ultrafine needles in the cubes, as typically shown in Figure 4b. After the reaction for 3 h, many concave nanocubes evolved into the chestnut-like nanocrystals (Figure 4c.). When the reaction time was up to 4 h, all concave nanocubes changed into the chestnut-like nanocrystals with high number density of nanoneedles, as shown in Figure 2. The longer reaction would only induce insignificant change in morphology, as typically demonstrated in Figure 4d. Correspondingly, the time-dependent optical absorbance spectra of the colloidal solutions showed a red-shift of the main absorption peak with the extension of reaction time (up to 4 h), from 700 nm to about 745 nm, in addition to the increasing peak intensity induced by the rising Au colloidal amount, as shown in Figure S1.

### 3.2. Influence Factors

The experiments have demonstrated that the formation of the chestnut-like Au nanocrystals were associated with the addition conditions of Au seed solution, including adding rate and amounts.

#### 3.2.1. Addition Rate of Au Seed Solution

**Figure 5:**
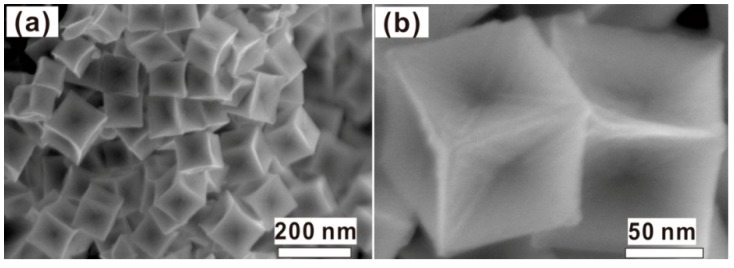
FESEM images of the products obtained by injecting 50 μL Au seeds into the growth solution at the rate of 10 μL/min. (**a**) Low and (**b**) high magnification.

It has been found that the adding rate of Au seeds could appreciably influence the morphology of Au nanocrystals. When a 50 μL diluted Au seed solution was, at a low rate (say, 10 μL/min), injected into the growth solution, the concave Au nanocubes with about 100 nm in the edge length were finally obtained, as illustrated in Figure 5a,b. They are completely different in morphology from those obtained at a moderate addition rate (about 25 μL /min) and shown in Figure 2. Obviously, such concave nanocubes are enclosed by {111} facets and [100] edges, as previously reported [16,27,28].

**Figure 6:**
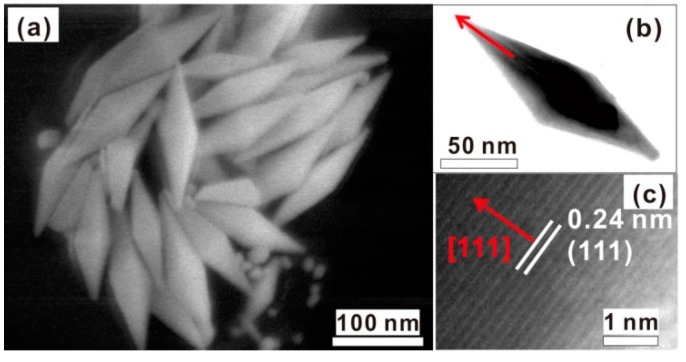
The morphology and microstructure of the products obtained by injecting the 50 μL Au seeds at the rate of 50 μL/min. (**a**) The FESEM image. (**b**) The TEM image of a single Au nanospindle. (**c**) The HRTEM image of (**b**) marked with an arrow. The arrows in (**b**,**c**) are parallel.

In contrast, if the 50 μL diluted Au seed solution was injected into the growth solution at a fast rate (say, 50 μL/min), Au nanospindles formed, as typically demonstrated in Figure 6a. Such nanospindles were about 150 nm in length, and the HRTEM examination has revealed that the long axis of the nanospindles was parallel to the [111] direction, as clearly shown in Figure 6b,c. By only adding the seeds at an appropriate or moderate rate (about 25 μL/min), could we fabricate the chestnut-like Au nanocrystals with high-density tips, as shown in Figure 2.

#### 3.2.2. The Amount of Au Seeds Solution

Moreover, we found that although the change in addition amount of the seed solution could not affect the morphology of Au nanostructure, it could change the size of the crystals. When the amount of added seed solution was reduced from 100 μL to 10 μL, keeping the addition rate of 25 μL/min, the products were mostly chestnut-like in morphology, but the mean sizes of the Au nanocrystals increased from 80 nm to 140 nm, as shown in Figure S2. Correspondingly, the SPR peak of the colloidal solutions was red-shifted from 642 nm to 805 nm, as demonstrated in Figure S3. This can be attributed to the increasing particle size [29]. Further, under a slow addition rate (say, 10 μL/min), concave nanocubes were formed (Figure 5), but the evolutions of the size and the SPR peak-position with the addition amount were similar to those above, as demonstrated in Figures S4 and S5.

### 3.3. Formation of Chestnut-Like Au Nanocrystals

**Figure 7:**
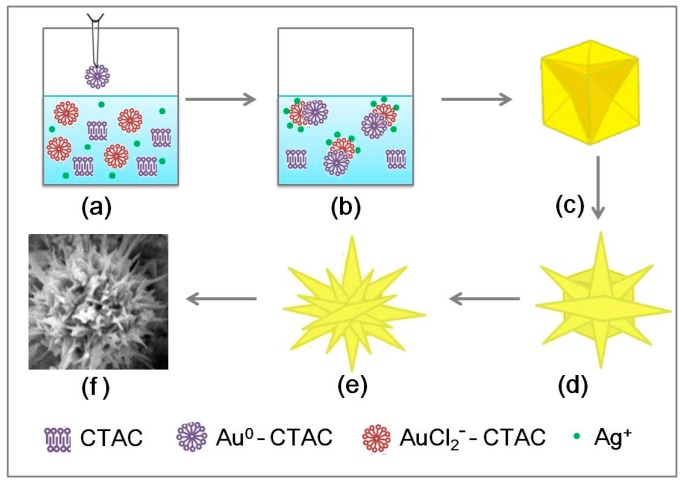
Schematic illustration for the formation of the chestnut-like Au nanocrystals. (**a**) The seeds were dropped into the growth solution. (**b**) AuCl_2_^−^ complex was reduced onto the surface of Au^0^ using electrons from ascorbic acid in the growth solution. (**c**) Formation of concave nanocubes during the initial growth period. (**d**) The radial growth of [111]-oriented nanoneedles. (**e**) The branched growth on the formed nanoneedles. (**f**) The chestnut-like Au nanocrystal.

Now, the formation of the chestnut-like Au nanocrystals will be discussed, as shown in Figure 7. In the seed solution, the AuCl_4_^−^ complex with CTAC to form AuCl_4_^−^-CTAC micelles, due to strong reduction capacity of NaBH_4_, Au^3+^ was reduced to Au^0^ directly, and obtained Au^0^-CTAC micelles [30,31,32,33].

AuCl_4_^−^ + 3e^−^→Au^0^ + 4Cl^−^ (1)

But in the growth solution, the reduction capacity of AA was weaker than NaBH_4_ [30,31,32,33], the Au^3+^ was reduced to Au^+^, and formed AuCl_2_^−^-CTAC micelles.

AuCl_4_^−^ + 2e^−^→AuCl_2_^−^ + 2Cl^−^ (2)

When Au seeds were added into the growth solution, the transport of bound Au ions to the growing seed particle was controlled by double-layer interaction of the cationic micelles with the micelle-coated Au seeds [31]. Moreover, in the presence of chloride ions, the addition of Ag^+^ slows down the growth rates on {110} facets, promoting the preferential deposition of Au^0^ at the (100) planes (the most energy favorable locations), therefore favoring the asymmetric growth in the [100] direction [34,35,36].

When a moderate rate (about 25 μL/min) of seeds were added, the magnitude of the seeds’ growth rate should also be moderate. In this case, the chestnut-like nanocystals would be formed, as schematically illustrated in Figure 7. After dropping the seed solution into the growth solution, the AuCl_2_^−^ complex was reduced onto the surface of Au^0^ using electrons from ascorbic acid, which induced the growth of the added seeds, as shown in Figure 7a,b. During the initial growth period (<1 h), the seeds gradually grew from the initial equi-axial shape into the concave nanocubes (Figure 7c), which were enclosed by the edges [100] and the facets {111}, as shown in Figure 5a. Based on moderate growth rate, the concave nanocubes evolved into nanocrystals built of the radial [111]-oriented nanoneedles, as shown in Figure 3d, with the reaction going on (Figure 7d). Further, more and more branches also grew along [111] at the defect sites on the pre-formed nanoneedles (Figure 7e). Finally, the chestnut-like Au nanocrystals (Figure 7f) with a high number density of tips were formed, as shown in Figure 2 and Figure 7f.

According to the above discussion, the reaction rate is crucial. Obviously, the slow reaction should be beneficial to the formation of concave nanocubes, due to the growth along both [111] and [100]. Otherwise, the fast reaction would only induce the nanoneedles along the [111]. The rate of adding seeds can determine reaction rate, and hence influence the growth kinetics under the given temperature. When a low rate (say, 10 μL/min) of Au seeds were added, the reaction rate was slow, and the Au seeds slowly grew along the [111] and [100], concave nanocubes form (Figure 5). In contrast, if the seed solution was added very fast (say, 50 μL/min), the reaction rate significantly increased. In this case, the Au seeds would grow fast and preferentially along [111], leading to the formation of the Au nanospindles, as shown in Figure 6.

We also studied the effect of the seeds’ amount, the results (Figures S2 and S4) showed an increase in size of nanostructures with decreasing amount of seeds, due to unchanged growth kinetics under the given addition rates. In addition, the increase in size resulted in red-shift of the SPR (Figures S3 and S5) [16].

### 3.4. The SERS Activity and SERS-Based Detection of Organochlorine Pesticides

As mentioned above, the chestnut-like Au nanocrystals are of a high number density of nanoneedles. Such nanocrystals should generate strong electromagnetic field enhancement at the tips and gaps among the nanoneedles, and present robust SERS performance [16,17,18,37]. Here, we used the 4-Aminothiophenol (4-ATP) as probe molecules (a well-known SERS analyte), and organochlorine pesticide lindane (γ-666) as the target molecules to study the SERS activity and SERS-based detection application of the chestnut-like Au nanocrystals.

#### 3.4.1. The Au Nanocrystals-Built Films for SERS Substrates

**Figure 8:**
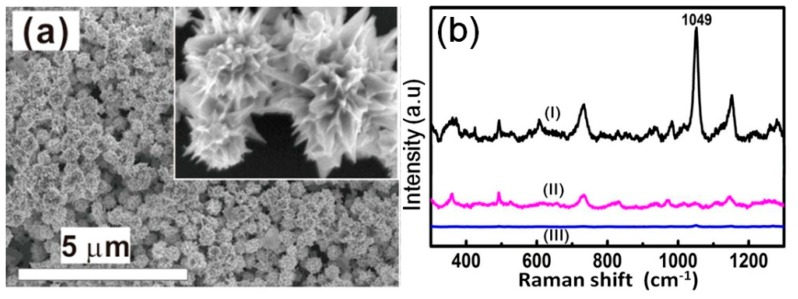
(**a**) The FESEM image of the as-prepared chestnut-like Au nanocrystals-built film (~500 nm in thickness) on a silicon substrate. The inset is the local magnified image. (**b**) The Raman spectra of the chestnut-like Au nanocrystals-built film after argon plasma bombardment for different times. Curves (I, II, III): after bombardment for 0 min, 5 min, and 10 min, respectively.

The films built of different Au nanocrystals were first prepared as the SERS substrates by dropping the solution on the Si substrate and drying, as displayed in Figure 8a and Figure S6. The absorption peaks of the colloidal solutions of nanospindles, concave nanocubes, and chestnut-like nanoparticles were located at 693, 721, and 738 nm, respectively, while those of their assembled films show a red shift to 762, 826, and 847 nm, respectively, due to the coupling effects, as demonstrated in Figure S7.

Since CTAC could be adsorbed on the Au nanocrystals due to the preparation conditions and interfere with the Raman signals of the target molecules, the assembled films were cleaned by argon plasma bombardment to remove the adsorbed species before use as the SERS substrate. It was found that the CTAC could be removed completely after bombardment for 10 min [38], as shown in Figure 8b. Further, the prepared nanocrystal-built films displayed high reproducibility (Figures S8 and S9). In addition, different samples with same morphology also had good reproducibility (Figure S10). As for the optimum excitation wavelength for these Au nanocrystal-built films, it was revealed that the excitation at 785 nm could induce stronger Raman signals than those at 532 nm and 633 nm, as illustrated in Figure S11. Finally, we studied the effect of film thickness. It was confirmed that when the film was thick enough (about 500 nm), the SERS signal no longer changed (Figure S12). On the basis of the above, the substrate with a 500 nm thickness was prepared after argon plasma bombardment for 10 min, and excitation at 785 nm was employed.

#### 3.4.2. Estimation of Enhancement Factor

**Figure 9:**
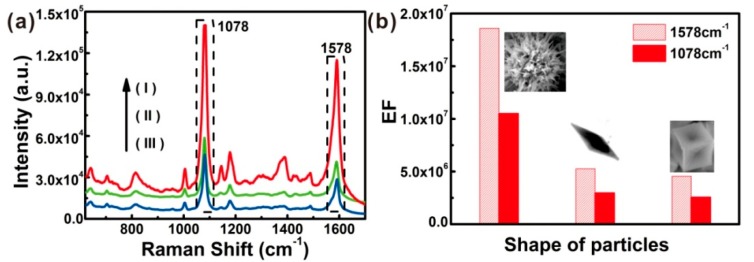
(**a**) The Raman spectra of the different substrates after dropping 20 μL 10^−5^ M 4-ATP ethanol solution and drying. The objective was 50× telephoto, laser power was 2 mW, and the acquisition time was 5 s. Curves (I), (II), and (III) correspond to the films built of the chestnut-like Au nanocrystals, the Au nanospindles, and the concave Au nanocubes, respectively. (**b**) Enhancement factors at the peaks at 1078 cm^−1^ and 1578 cm^−1^ for the different Au nanocrystal-built films.

Figure 9a displays the SERS spectra of different Au substrates of 10^−5^ M 4-ATP ethanol solution (20 μL). The SERS spectral peaks coincide with solid intrinsic spectrum of 4-ATP (Figure S13a). Two main peaks at 1078 cm^−1^ and 1578 cm^−1^ were attributed to the C−S stretching mode and phenol ring C−C stretching mode, respectively [39]. Comparing the main intensities of three SERS spectra with that of the solid 4-ATP Raman peaks, great enhancement was revealed. Moreover, the enhance effect of chestnut-like substrate was the strongest, while Au nanocubes and Au nanospindles demonstrated lower enhancement.

For quantitative analysis of the SERS activity or effect for these Au nanocrystals, enhancement factor (EF) was calculated in accordance with Formula (4) [40,41,42]:

(4) $$\mathrm{EF}=\frac{I_{\mathrm{SERS}}/N_{\mathrm{SERS}}}{I_{\mathrm{RS}}/N_{\mathrm{RS}}}$$

where I_RS_ and I_SERS_ are the intensities of 4-ATP main peaks (1078 cm^−1^ and 1578 cm^−1^) without (Si substrate) and with (Au nanostructure) enhancement, respectively. N_RS_ and N_SERS_ are the numbers of different concentrations (50 μL 0.1M and 20 μL 10^−5^ M) 4-ATP within φ1 μm laser spot areas on the films. Here, the silicon size was about 3 mm × 3 mm, and the diameter of the Au nanocrystals’ circular area was about φ3 mm. The obtained spectra are shown in Figure S13b and Figure 9a, respectively. The calculated values of N_RS_ and N_SERS_ are 3.4 × 10^11^ and 1.4 × 10^7^. And the calculated EF values of different Au nanostructure in accordance with Formula (4) at 1078 cm^−1^ and 1578 cm^−1^, as shown in Figure 9b. The results revealed that chestnut-like Au nanostructure displayed the strongest SERS enhancement, and EF values were higher than 10^7^, while the concave nanocubes and nanospindles showed much lower EF values.

The highest enhancement factor can be attributed to the special structure of the chestnut-like Au nanocrystals. The chestnut-like nanocrystal-built film possesses the much higher number density of tips than that of the nanocubes and nanospindle films, as clearly illustrated in Figure S6. The sharp nanoneedles would generate significant electromagnetic field enhancement under the excitation of an external field, due to the tip-effect [33,43]. Moreover, nanogaps on the multi-branch structures could be stimulated and induced lots of “hot spots”, and generate robust electromagnetic field enhancement [44,45,46,47].

#### 3.4.3. Application in Organochlorine Pesticide Detection

It is well known that organochlorine pesticides belong to highly-toxic substances [48,49]. Among them, lindane (or γ-666) is the most typical and toxic organochlorine pesticide. They are commonly detected using some conventional techniques, including the gas chromatography mass spectrometry (GC-MS) coupling technique, and gas chromatography/electron capture detection (GC/ECD) [50,51]. These analysis methods are off-site and time-consuming. The SERS-based technique could be a promising method for the fast and sensitive detection of toxic molecules [52,53,54]. For instance, Kubackova et al. [54] used the alkyl dithiol-functionalized metal nanoparticles as a SERS substrate to detect lindane molecules (or γ-666), which are the most typical and toxic organochlorine pesticides, with the detection limit of 1 ppm. In our previous work [33], the concave trisoctahedral and calyptriform Au nanocrystals were used as the SERS substrates for lindane detection, and the detection limit was down to 30 ppb. However, the detection limit is still relatively high, and especially, the identification of the organochlorine isomers has not been involved.

**Figure 10:**
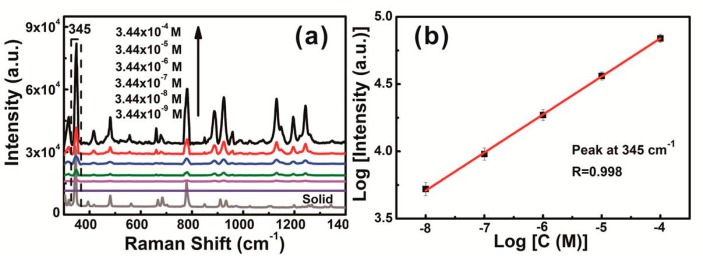
(**a**) The surface enhanced Raman scattering (SERS) spectra of γ-666 obtained from chestnut-like Au nanocrystal films, the concentrations varying from 3.44 × 10^−4^ M to 3.44 × 10^−9^ M, and drying. The bottom curve is the spectrum of solid γ-666. The objective was 50× telephoto, laser power was 2 mW, and the acquisition time was 5 s. (**b**) The relationship between SERS intensity *I* at 345 cm^−1^ and concentration *C* meets the double logarithm linear. *R* is the correlation coefficient.

Here, we chose lindane as the target molecule. The above mentioned chestnut-like Au nanocrystal-built film with the highest EF value was used to prove SERS-based detection of organochlorine molecules. The relationship between concentration and Raman intensity of γ-666 was thus measured. Figure 10a displays the SERS spectra of the film after soaking for 8 h in γ-666 ethanol solutions, the concentrations varying from 3.44 × 10^−4^ M (100 ppm) to 3.44 × 10^−9^ M (1 ppb), and drying. The SERS spectral peaks coincided with solid intrinsic spectrum of γ-666 (see the bottom curve in Figure 10a). One of the main characteristic peaks at 345 cm^−1^ is ascribed to the stretching vibration of C−Cl in γ-666, and the bands in the region of 400–1400 cm^−1^ stand for C−C and C−H stretching, and CH_2_ bending from the aliphatic cyclic structure of the analyzed molecules [54,55,56].

From the Figure 10a, we can clearly see that SERS intensity decreases with decreasing concentration, and the detection limit concentration was down to about 10 ppb. So, such chestnut-like Au nanocrystal-built film could be a good candidate for the SERS substrate for trace recognition of γ-666 in solution. Moreover, the relationship between SERS intensity *I* and concentration *C* is in line with the double logarithm linear, as presented in Figure 10b. Here, the main peak 345 cm^−1^ is an example. This linear relationship meets Freundlich-typed adsorption of the γ-666 on the Au nanostructure, as previously reported [33,57]. We could, thus, realize the quantitative SERS-based detection in a large concentration range.

**Figure 11:**
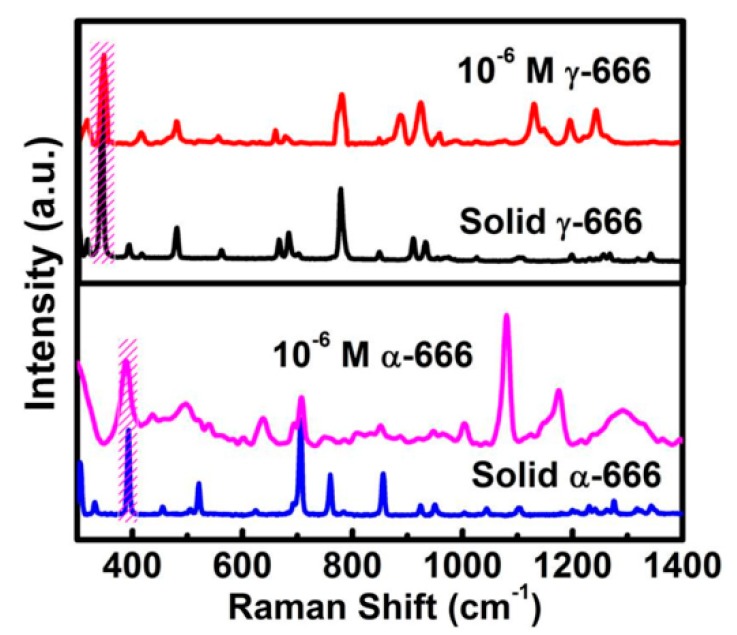
The Raman spectra of the pure solid γ-666 and α-666, and the SERS spectra of γ-666 and α-666 molecules on the chestnut-like Au nanocrystals’ film after soaking in 10^−6^ M solutions (excited at 785 nm).

Finally, it should be mentioned that the lindane’s isomers could also be differentiated using the chestnut-like Au nanocrystals for the SERS substrate, as typically shown in Figure 11, corresponding to the Raman spectra of α-666 and γ-666 molecules on the chestnut-like Au nanocrystal-built film. Despite their similar molecule’s structure, they exhibit different Raman patterns from each other. This is attributed to the fact that there are significant differences between α-666 and γ-666 molecules in the vibrations of C–Cl and C–C bonds, and the internal and external of ring [54], and the Raman signals are very sensitive to the molecular vibrations.

## 4. Conclusions

In summary, we have presented a modified seed-growth approach to construct the Au configuration with high-density tips based on kinetically-controlled seed growth via adjusting the addition rate of Au seeds into the growth solution. Under a proper addition rate of seeds, which could determine growth kinetics of crystal nuclei, the chestnut-like Au nanocrystals with high-density tips about 100 nm in size were obtained. The formation of such configurations is ascribed to the priority growth of seeds along [111], which induces the concave nanocubes in the initial growth period (<1 h) and the nanocrystals built of the high-density radial [111]-oriented nanoneedles in the final stage. The addition amount of seeds could determine the size of final nanocrystals, but only insignificantly change the morphology due to the unchanged growth kinetics under the given addition rates. Moreover, such chestnut-like Au nanocrystal-built film with high-density tips have displayed the powerful SERS performance (EF > 10^7^). When it is used for the SERS-based detection of the γ-666 molecules, the limit of detection was about 10 ppb. The relationship between SERS intensity *I* and concentration *C* is in line with the double logarithm linear, and accords with Freundlich-typed adsorption. This work presents a simple approach to the Au nanocrystals with high-density tips, and provides a highly efficacious SERS-substrate for the quantitative and trace recognition of toxic chlorinated pesticides.

## Supplementary Materials

The following are available online at http://www.mdpi.com/2079-4991/8/7/560/s1. SEM images, size distribution histograms and optical absorbance spectra of nanocrystals obtained under different amounts of seed solution; SEM images of different nanocrystal-built films; Raman spectra of 20 random spots on the different nanocrystals-built films; the effects of films’ thickness and excitation wavelength; and Raman spectra of solid and 0.1 M 4-ATP.

## References

[1] Jin Q.R., Li M., Polat B., Paidi S.K., Dai A., Zhang A., Pagaduan J.V., Barman I., Gracias D.H. (2017). Mechanical Trap Surface-Enhanced Raman Spectroscopy for Three-Dimensional Surface Molecular Imaging of Single Live Cells. Angew. Chem. Int. Ed..

[2] Zhou Q.F., Zheng J., Qing Z.H., Zheng M.J., Yang J.F., Yang S., Ying L., Yang R.H. (2016). Detection of Circulating Tumor DNA in Human Blood via DNA-Mediated Surface-Enhanced Raman Spectroscopy of Single-Walled Carbon Nanotubes. Anal. Chem..

[3] Zaleski S., Clark K.A., Smith M.M., Eilert J.Y., Doty M., Van Duyne R.P. (2017). Identification and Quantification of Intravenous Therapy Drugs Using Normal Raman Spectroscopy and Electrochemical Surface Enhanced Raman Spectroscopy. Anal. Chem..

[4] Zhou B.B., Li S.F., Tang X.H., Li P., Cao X.M., Yu B.R., Yang L. B., Liu J.H. (2017). Real-time Monitoring of Plasmon-induced Proton Transfer of Hypoxanthine in Serum. Nanoscale.

[5] Ben-Jaber S., Peveler W.J., Quesada-Cabrera R., Cortes E., Sotelo-Vazquez C., Abdul-Karim N., Maier S.A., Parkin I.P. (2016). Photo-induced Enhanced Raman Spectroscopy for Universal Ultra-trace Detection of Explosives, Pollutants and Biomolecules. Nat. Commun..

[6] Cui L., Zhang Y.J., Huang W.E., Zhang B.F., Martin F.L., Li J.Y., Zhang K.S., Zhu Y.G. (2016). Surface-Enhanced Raman Spectroscopy for Identification of Heavy Metal Arsenic(V)-Mediated Enhancing Effect on Antibiotic Resistance. Anal. Chem..

[7] Wang P., Wu L., Lu Z.C., Li Q., Yin W.M., Ding F., Han H.Y. (2017). Gecko-Inspired Nanotentacle Surface-Enhanced Raman Spectroscopy Substrate for Sampling and Reliable Detection of Pesticide Residues in Fruits and Vegetables. Anal. Chem..

[8] Craig A.P., Franca A.S., Irudayaraj J. (2013). Surface-Enhanced Raman Spectroscopy Applied to Food Safety. Annu. Rev. Food. Sci. Techonl..

[9] Xu L.G., Zhao S., Ma W., Wu X.L., Li S., Kuang H., Wang L.B., Xu C.L. (2016). Multigaps Embedded Nanoassemblies Enhance In Situ Raman Spectroscopy for Intracellular Telomerase Activity Sensing. Adv. Funct. Mater..

[10] Henry A.I., Sharma B., Cardinal M.F., Kurouski D., Van Duyne R.P. (2016). Surface-Enhanced Raman Spectroscopy Biosensing: In Vivo Diagnostics and Multimodal Imaging. Anal. Chem..

[11] Zhou B., Li X., Tang X., Li P., Yang L., Liu J. (2017). Highly Selective and Repeatable Surface-Enhanced Resonance Raman Scattering Detection for Epinephrine in Serum based on Interface Self-Assembled 2D Nanoparticles Arrays. ACS Appl. Mater. Interfaces.

[12] Pazos-Perez N., Barbosa S., Rodriguez-Lorenzo L., Aldeanueva-Potel P., Perez-Juste J., Pastoriza-Santos I., Alvarez-Puebla R.A., Liz-Marzan L.M. (2010). Growth of Sharp Tips on Gold Nanowires leads to Increased Surface-Enhanced Raman Scattering Activity. J. Phys. Chem. Lett..

[13] Pradhan M., Chowdhury J., Sarkar S., Sinha A.K., Pal T. (2012). Hierarchical Gold Flower with Sharp Tips from Controlled Galvanic Replacement Reaction for High Surface Enhanced Raman Scattering Activity. J. Phys. Chem. C.

[14] Lee J., Hua B., Park S., Ha M., Lee Y., Fan Z., Ko H. (2014). Tailoring Surface Plasmons of High-Density Gold Nanostar Assemblies on Metal Films for Surface-Enhanced Raman Spectroscopy. Nanoscale.

[15] Liu D.Q., Wang X., He D.Y., Dao T.D., Nagao T., Weng Q.H., Tang D.M., Wang X.B., Tian W., Golberg D. (2014). Magnetically Assembled Ni@Ag Urchin-Like Ensembles with Ultra-Sharp Tips and Numerous Gaps for SERS Applications. Small.

[16] Zhang Q., Large N., Wang H. (2014). Gold Nanoparticles with Tipped Surface Structures as Substrates for Single-Particle Surface-Enhanced Raman Spectroscopy: Concave Nanocubes, Nanotrisoctahedra, and Nanostars. ACS Appl. Mater. Interfaces.

[17] Fang J., Du S., Lebedkin S., Li Z., Kruk R., Kappes M., Hahn H. (2010). Gold Mesostructures with Tailored Surface Topography and Their Self-Assembly Arrays for Surface-Enhanced Raman Spectroscopy. Nano Lett..

[18] Liu Z., Yang Z., Peng B., Cao C., Zhang C., You H., Xiong Q., Li Z., Fang J. (2014). Highly Sensitive, Uniform, and Reproducible Surface-Enhanced Raman Spectroscopy from Hollow Au-Ag Alloy Nanourchins. Adv. Mater..

[19] Kumar P.S., Pastoriza-Santos I., Rodriguez-Gonzalez B., Garcia de Abajo F.J., Liz Marzan L.M. (2008). High-Yield Synthesis and Optical Response of Gold Nanostars. Nanotechnology.

[20] Barbosa S., Agrawal A., Rodriguez-Lorenzo L., Pastoriza-Santos I., Alvarez-Puebla R.A., Kornowski A., Weller H., Liz-Marzan L.M. (2010). Tuning Size and Sensing Properties in Colloidal Gold Nanostars. Langmuir.

[21] Lu F., Zhang Y., Zhang L., Zhang Y., Wang J.X., Adzic R.R., Stach E.A., Gang O. (2011). Truncated Ditetragonal Gold Prisms as Nanofacet Activators of Catalytic Platinum. J. Am. Chem. Soc..

[22] Lu F., Tian Y., Liu M., Su D., Zhang H., Govorov A.O., Gang O. (2013). Discrete Nanocubes as Plasmonic Reporters of Molecular Chirality. Nano Lett..

[23] Lu F., Yager K.G., Zhang Y., Xin H., Gang O. (2015). Superlattices Assembled Through Shape-Induced Directional Binding. Nat. Commun..

[24] Ma C., Gao Q.Q., Hong W., Fan J., Fang J.X. (2017). Real-Time Probing Nanopore-in-Nanogap Plasmonic Coupling Effect on Silver Supercrystals with Surface-Enhanced Raman Spectroscopy. Adv. Funct. Mater..

[25] Tian C.F., Li J., Ma C.S., Wang P., Sun X.H., Fang J.X. (2015). An Ordered Mesoporous Ag Superstructure Synthesized via a Template Strategy for Surface-Enhanced Raman Spectroscopy. Nanoscale.

[26] Cheng L., Ma C.S., Yang G., You H.J., Fang J.X. (2014). Hierarchical Silver Mesoparticles with Tunable Surface Topographies for Highly Sensitive Surface-Enhanced Raman Spectroscopy. J. Mater. Chem. A.

[27] Xia X.H., Zeng J., McDearmon B., Zheng Y.Q., Li Q.G., Xia Y.N. (2011). Silver Nanocrystals with Concave Surfaces and Their Optical and Surface-Enhanced Raman Scattering Properties. Angew. Chem. Int. Ed..

[28] Zhang J.A., Langille M.R., Personick M.L., Zhang K., Li S.Y., Mirkin C.A. (2010). Concave Cubic Gold Nanocrystals with High-Index Facets. J. Am. Chem. Soc..

[29] Rycenga M., Langille M.R., Personick M.L., Ozel T., Mirkin C.A. (2012). Chemically Isolating Hot Spots on Concave Nanocubes. Nano Lett..

[30] Personick M.L., Langille M.R., Zhang J., Mirkin C.A. (2011). Shape Control of Gold Nanoparticles by Silver Underpotential Deposition. Nano Lett..

[31] Perez-Juste J., Liz-Marzan L.M., Carnie S., Chan D.Y.C., Mulvaney P. (2004). Electric-Field-Directed Growth of Gold Nanorods in Aqueous Surfactant Solutions. Adv. Funct. Mater..

[32] Murphy C.J., San T.K., Gole A.M., Orendorff C.J., Gao J.X., Gou L., Hunyadi S.E., Li T. (2005). Anisotropic Metal Nanoparticles: Synthesis, Assembly, and Optical Applications. J. Phys. Chem. B.

[33] Zhou X., Zhao Q., Liu G., Zhang H., Li Y., Cai W. (2017). Temperature Regulation Growth of Au Nanocrystals: From Concave Trisoctahedron to Dendritic Structures and Their Ultrasensitive SERS-Based Detection of Lindane. J. Mater. Chem. C.

[34] Jessl S., Tebbe M., Guerrini L., Fery A., Alvarez-Puebla R.A., Pazos-Perez N. (2018). Silver-Assisted Synthesis of Gold Nanorods: The Relation between Silver Additive and Iodide Impurities. Small.

[35] Tebbe M., Kuttner C., Mayer M., Maennel M., Pazos-Perez N., Konig T.A.F., Fery A. (2015). Silver-Overgrowth-Induced Changes in Intrinsic Optical Properties of Gold Nanorods: From Noninvasive Monitoring of Growth Kinetics to Tailoring Internal Mirror Charges. J. Phys. Chem. C.

[36] Mayer M., Scarabelli L., March K., Altantzis T., Tebbe M., Kociak M., Bals S., de Abajo F.J.G., Fery A., Liz-Marzan L.M. (2015). Controlled Living Nanowire Growth: Precise Control over the Morphology and Optical Properties of AgAuAg Bimetallic Nanowires. Nano Lett..

[37] Li C.C., Shuford K.L., Park Q.H., Cai W.P., Li Y., Lee E.J., Cho S.O. (2007). High-Yield Synthesis of Single-Crystalline Gold Nano-Octahedra. Angew. Chem. Int. Ed..

[38] Liu G., Cai W., Kong L., Duan G., Li Y., Wang J., Zuo G., Cheng Z. (2012). Standing Ag nanoplate-built hollow microsphere arrays: Controllable structural parameters and strong SERS performances. J. Mater. Chem..

[39] Fang Y.R., Li Y.Z., Xu H.X., Sun M.T. (2010). Ascertaining p,p′-Dimercaptoazobenzene Produced from p-Aminothiophenol by Selective Catalytic Coupling Reaction on Silver Nanoparticles. Langmuir.

[40] McLean T.M., Cleland D., Gordon K.C., Telfer S.G., Waterland M.R. (2011). Raman Spectroscopy of Dipyrrins: Nonresonant, Resonant and Surface-Enhanced Cross-Sections and Enhancement Factors. J. Raman Spectrosc..

[41] Kleinman S.L., Sharma B., Blaber M.G., Henry A.I., Valley N., Freeman R.G., Natan M.J., Schatz G.C., Van Duyne R.P. (2013). Structure Enhancement Factor Relationships in Single Gold Nanoantennas by Surface-Enhanced Raman Excitation Spectroscopy. J. Am. Chem. Soc..

[42] Lee S.J., Morrill A.R., Moskovits M. (2006). Hot Spots in Silver Nanowire Bundles for Surface-Enhanced Raman Spectroscopy. J. Am. Chem. Soc..

[43] Camden J.P., Dieringer J.A., Zhao J., Van Duyne R.P. (2008). Controlled Plasmonic Nanostructures for Surface-Enhanced Spectroscopy and Sensing. Acc. Chem. Res..

[44] He X., Yue C., Zang Y.S., Yin J., Sun S.B., Li J., Kang J.Y. (2013). Multi-Hot Spot Configuration on Urchin-Like Ag Nanoparticle/ZnO Hollow Nanosphere Arrays for Highly Sensitive SERS. J. Mater. Chem. A.

[45] Butburee T., Bai Y., Pan J., Zong X., Sun C.H., Liu G., Wang L.Z. (2014). Step-Wise Controlled Growth of Metal@TiO_2_ Core-Shells with Plasmonic Hot Spots and Their Photocatalytic Properties. J. Mater. Chem. A.

[46] Jang S.H., Yoon J.H., Huh Y.D., Yoon S. (2014). Creating SERS Hot Spots on Ultralong Single-Crystal Beta-AgVO_3_ Microribbons. J. Mater. Chem. C.

[47] Li P., Yan X.N., Zhou F., Tang X.G., Yang L.B., Liu J.H. (2017). A Capillary Force-Induced Au Nanoparticle-Ag Nanowire Single Hot Spot Platform for SERS Analysis. J. Mater. Chem. C.

[48] Lopez-Tocon I., Otero J.C., Arenas J.F., Garcia-Ramos J.V., Sanchez-Cortes S. (2011). Multicomponent Direct Detection of Polycyclic Aromatic Hydrocarbons by Surface-Enhanced Raman Spectroscopy Using Silver Nanoparticles Functionalized with the Viologen Host Lucigenin. Anal. Chem..

[49] Peluso F., Dubny S., Othax N., Castelain J.G. (2014). Environmental Risk of Pesticides: Applying the DelAzulPestRisk Model to Freshwaters of an Agricultural Area of Argentina. Hum. Ecol. Risk Assess..

[50] Xu X.Q., Yang H.G., Li Q.L., Yang B.J., Wang X.R., Lee F.S.C. (2007). Residues of Organochlorine Pesticides in Near Shore Waters of LaiZhou Bay and JiaoZhou Bay, Shandong Peninsula, China. Chemosphere.

[51] Ali M., Kazmi A.A., Ahmed N. (2014). Study on Effects of Temperature, Moisture and pH in Degradation and Degradation Kinetics of Aldrin, Endosulfan, Lindane Pesticides During Full-Scale Continuous Rotary Drum Composting. Chemosphere.

[52] Liang X., Wang Y.S., You T.T., Zhang X.J., Yang N., Wang G.S., Yin P.G. (2017). Interfacial Synthesis of a Three-Dimensional Hierarchical MoS_2_-NS@Ag-NP Nanocomposite as a SERS Nanosensor for Ultrasensitive Thiram Detection. Nanoscale.

[53] Qi L., Xiao M.S., Wang F., Wang L.H., Ji W., Man T.T., Aldalbahi A., Khan M.N., Periyasami G., Rahaman M. (2017). Poly-Cytosine-Mediated Nanotags for SERS Detection of Hg^2+^. Nanoscale.

[54] Kubackova J., Fabriciova G., Miskovsky P., Jancura D., Sanchez-Cortes S. (2015). Sensitive Surface-Enhanced Raman Spectroscopy (SERS) Detection of Organochlorine Pesticides by Alkyl Dithiol-Functionalized Metal Nanoparticles-Induced Plasmonic Hot Spots. Anal. Chem..

[55] Guerrini L., Aliaga A.E., Carcamo J., Gomez-Jeria J.S., Sanchez-Cortes S., Campos-Vallette M.M., Garcia-Ramos J.V. (2008). Functionalization of Ag Nanoparticles with the Bis-acridinium Lucigenin as a Chemical Assembler in the Detection of Persistent Organic Pollutants by Surface-Enhanced Raman Scattering. Anal. Chim. Acta.

[56] Mishra S., Vallet V., Poluyanov L.V., Domcke W. (2006). Calculation of the Vibronic Structure of the Photodetachment Spectra of CCCl- and CCBr. J. Chem. Phys..

[57] Zhao Q., Liu G., Zhang H., Zhou F., Li Y., Cai W. (2017). SERS-based Ultrasensitive Detection of Organophosphorus Nerve Agents via Substrate’s Surface Modification. J. Hazard. Mater..

