# Supplementary material for: Kinetically-Controlled Growth of Chestnut-Like Au Nanocrystals with High-Density Tips and Their High SERS Performances on Organochlorine Pesticides
Source: Nanomaterials (Basel). 2018 Jul 23;8(7):560. doi: 10.3390/nano8070560 (PMC6071147; doi:10.3390/nano8070560)
Supplement: Supplementary file 1 [file nanomaterials-08-00560-s001.pdf]

Supporting Information

Kinetically Controlled Growth of Chestnut-like Au Nanocrystals with High-density Tips and their High SERS Performances to Organochlorine Pesticides

Xia Zhou[1,2,3], Qian Zhao[1] , Guangqiang Liu[1*], Hongwen Zhang[1], Yue Li[1], and Weiping Cai[1,2*]

[1] Key Lab of Materials Physics, Anhui Key Lab of Nanomaterials and Nanotechnology, Institute of Solid State Physics, Chinese Academy of Sciences, Hefei 230031, PR China.

[2] University of Science and Technology of China, Hefei 230026, PR China.

[3] School of Chemistry and Chemical Engineering, Suzhou University, Suzhou, Anhui 234000, PR China.

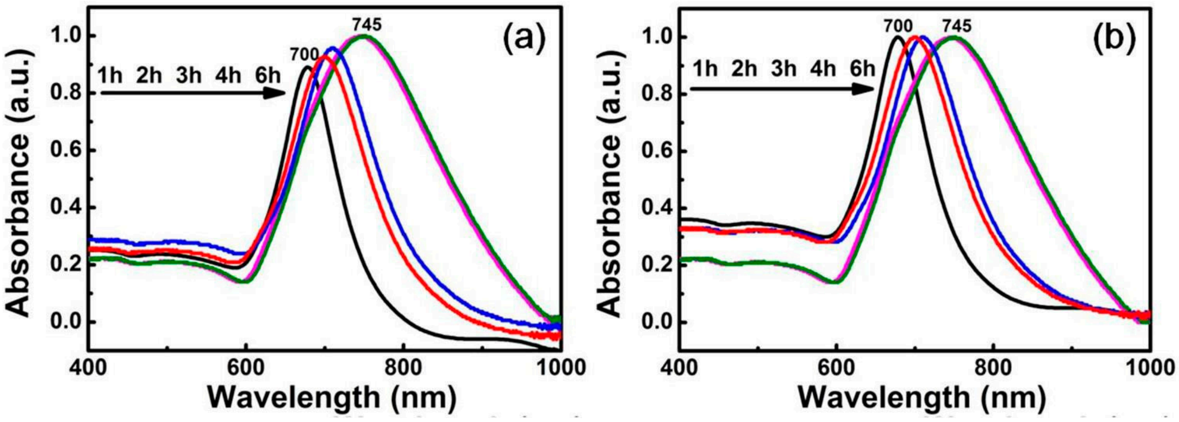

Figure S1. The optical absorbance spectra of the Au colloidal solutions obtained after adding the Au seeds into the growth solution for different durations. The spectra without (a) and (b) with normalizing, respectively.

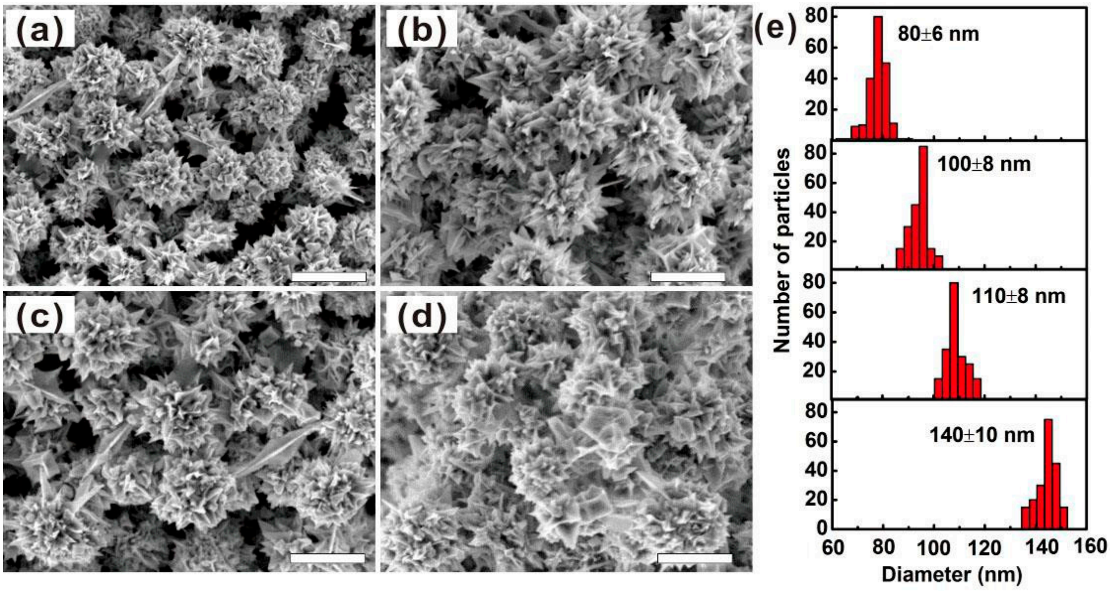

Figure S2. FESEM images of the products obtained by adding (a) 100 μL, (b) 50 μL, (c) 25 μL and (d) 10 μL of the diluted Au seed solution into the growth solution, at a moderate addition rate (25 μL/min). (e) The size distribution histograms of the chestnut-like Au nanocrystals. The panels from top to bottom correspond to (a−d). The scale bar is 100 nm.

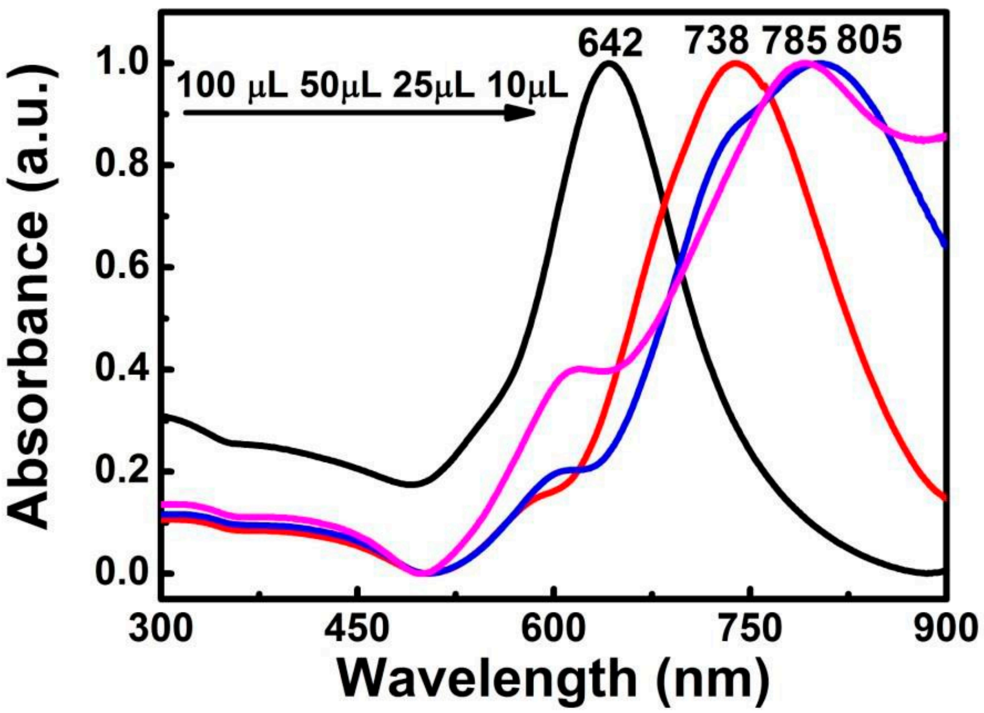

Figure S3. The optical absorbance spectra of the colloidal solutions obtained by adding the different amounts of the diluted Au seed solution into the growth solution at a moderate addition rate (25 μL/min).

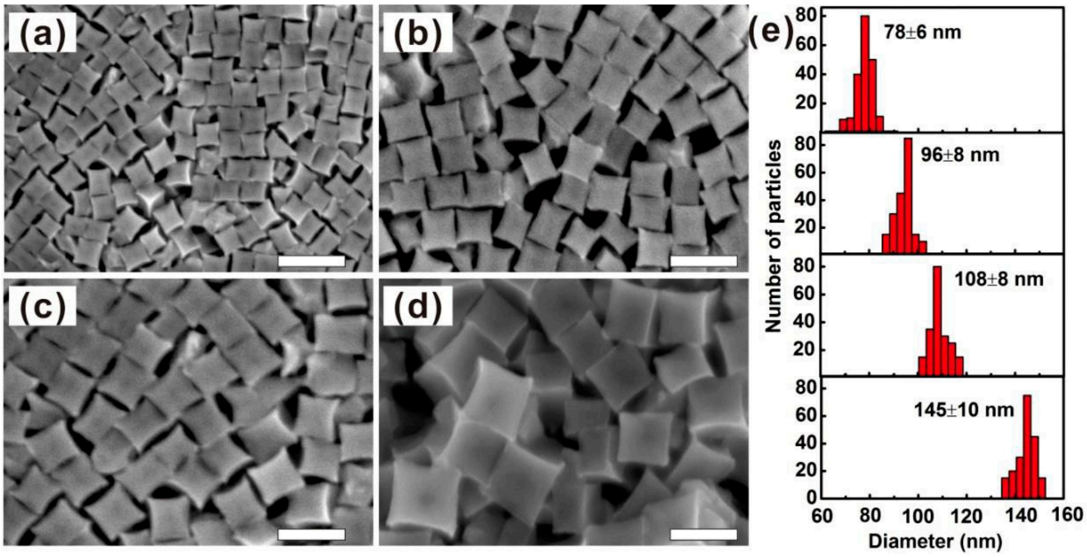

Figure S4. FESEM images of the products obtained by adding (a) 100μL, (b) 50μL, (c) 25μL and (d) 10 μL of the diluted Au seed solution into the growth solution at a slow addition rate (10 μL/min). (e) The size distribution histograms of the Au concave nanocubes. The panels from top to bottom correspond to (a–d). The scale bar is 200 nm.

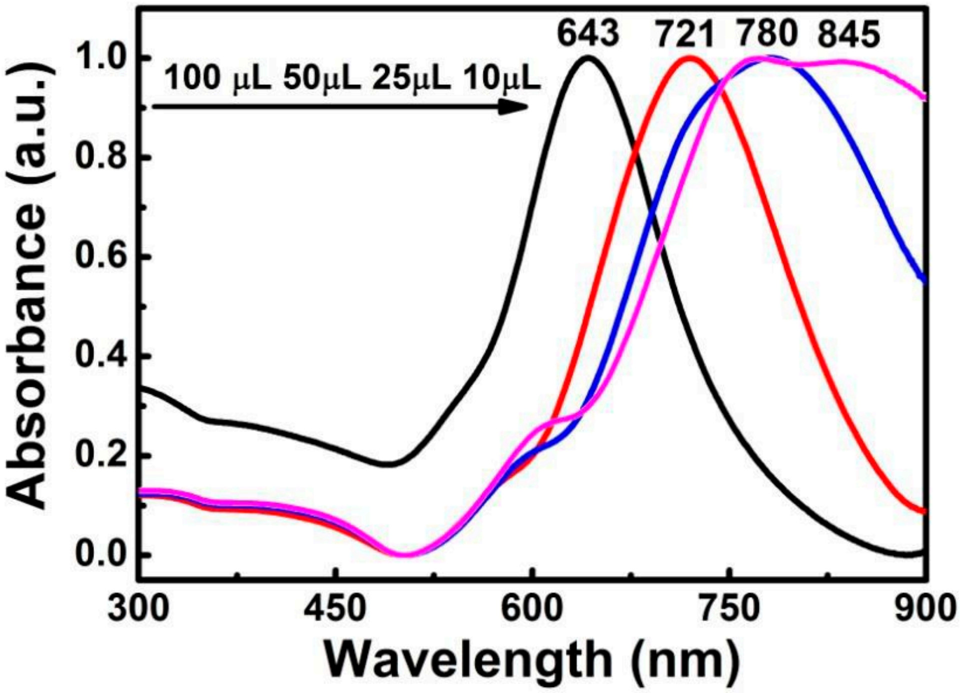

Figure S5. The optical absorbance spectra of the colloidal solutions obtained by adding the different amounts of the diluted Au seed solution into the growth solution at a slow addition rate (10 μL/min).

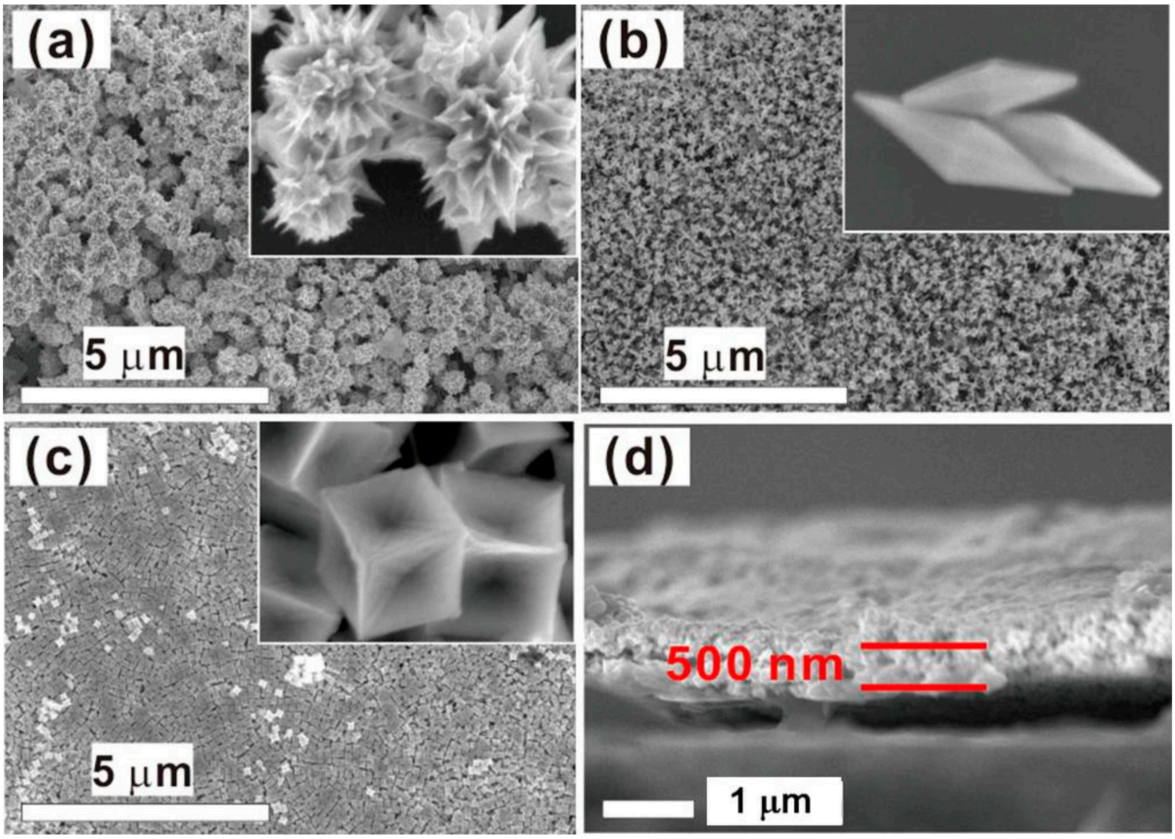

Figure S6. The FESEM images of the films built of (a) the chestnut-like Au nanocrystals, (b) the Au nanospindles and (c) the concave Au nanocubes. (d) The cross sectional image of the film in (a). The insets are the corresponding local magnified images.

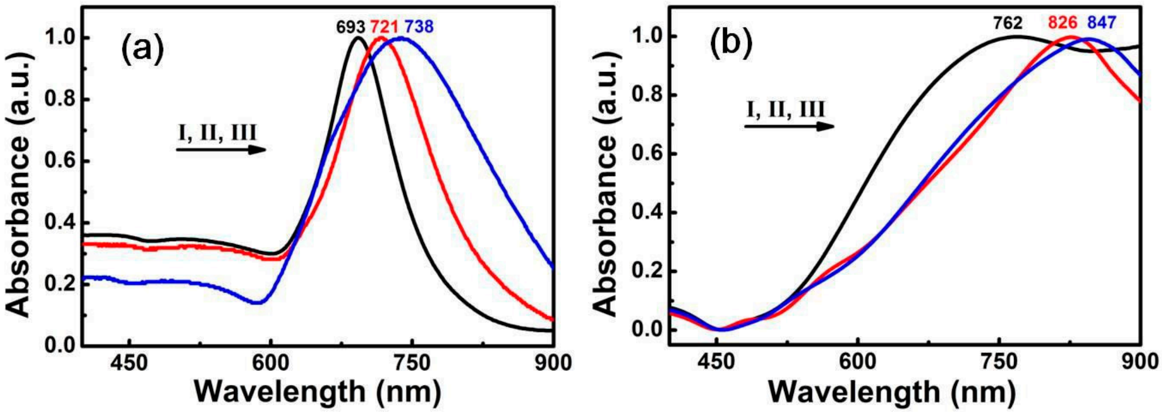

Figure S7. The optical absorbance spectra of (a) colloidal solutions and (b) assembled particles' films used for the SERS substrates. (I) Nanospindles, (II) Concave nanocubes and (III) Chestnut-like nanoparticles.

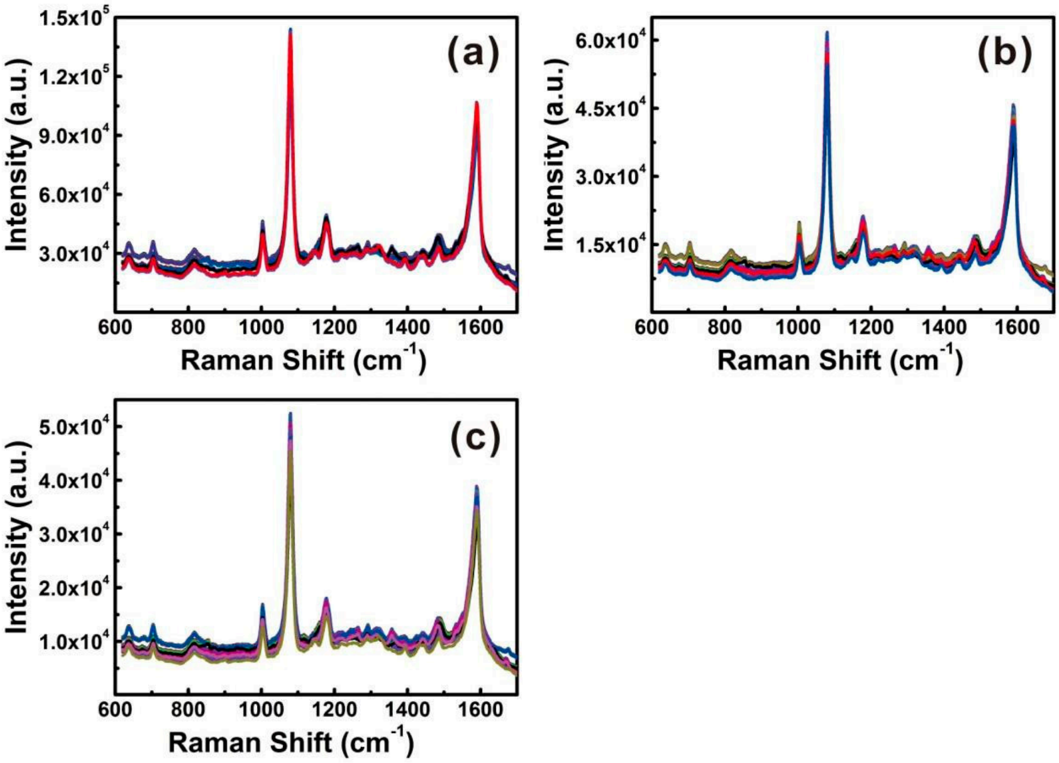

Figure S8. The Raman spectra from the 20 random spots on the films built of (a) the chestnut-like Au nanocrystals, (b) the Au nanospindles and (c) the concave Au nanocubes, after soaking in the solution with 10^{-5} M 4-ATP solution and drying (excited at 785nm).

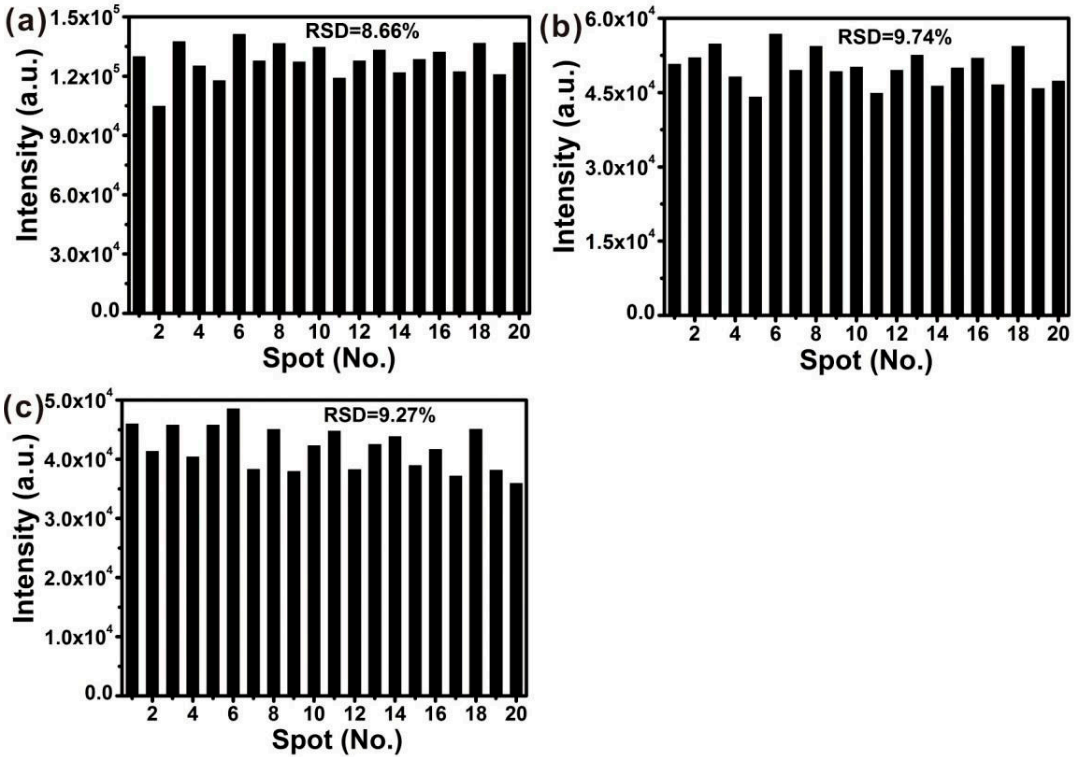

Figure S9. The intensity of the main peak at 1078 cm^{-1} from each spot on the Au nanocrystals-built films. The data in (a), (b) and (c) are from (a), (b) and (c) in Figure S6, respectively. RSD is the relative standard deviation of the intensity at 1078 cm^{-1}.

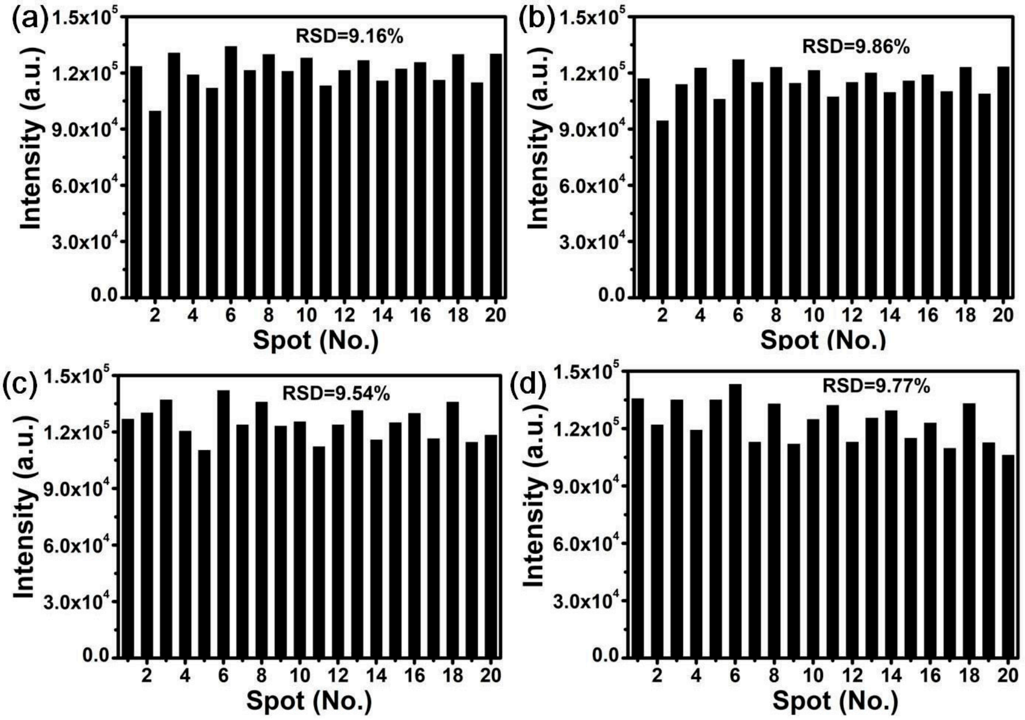

Figure S10. The intensity of the main vibration of 4-ATP spectra of the 20 spots from the chestnut-like Au nanocrystals built-films obtained at 4 different samples. The RSD of major peaks at 1078 cm^{-1} is used to estimate.

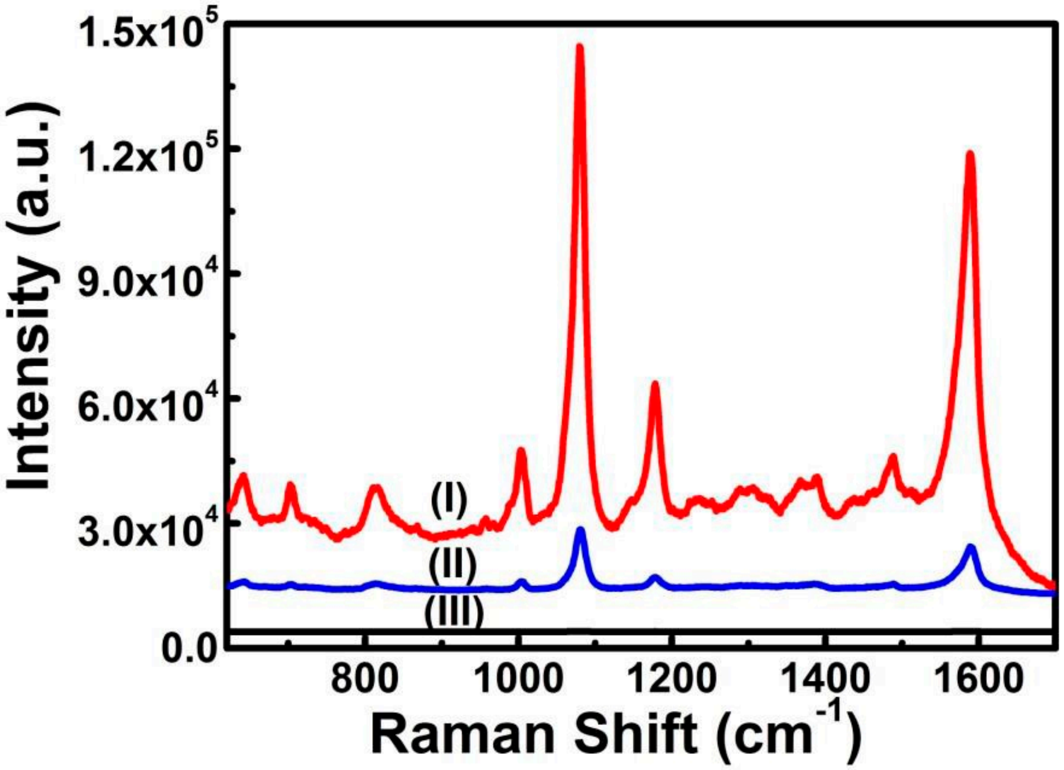

Figure S11. The Raman spectra of the chestnut-like Au nanocrystals-built film after soaking in the 4-ATP solution with 10^{-5} M and exciting at different wavelengths. (I) 785 nm; (II) 633 nm; (III) 532 nm. The integration time is 2s.

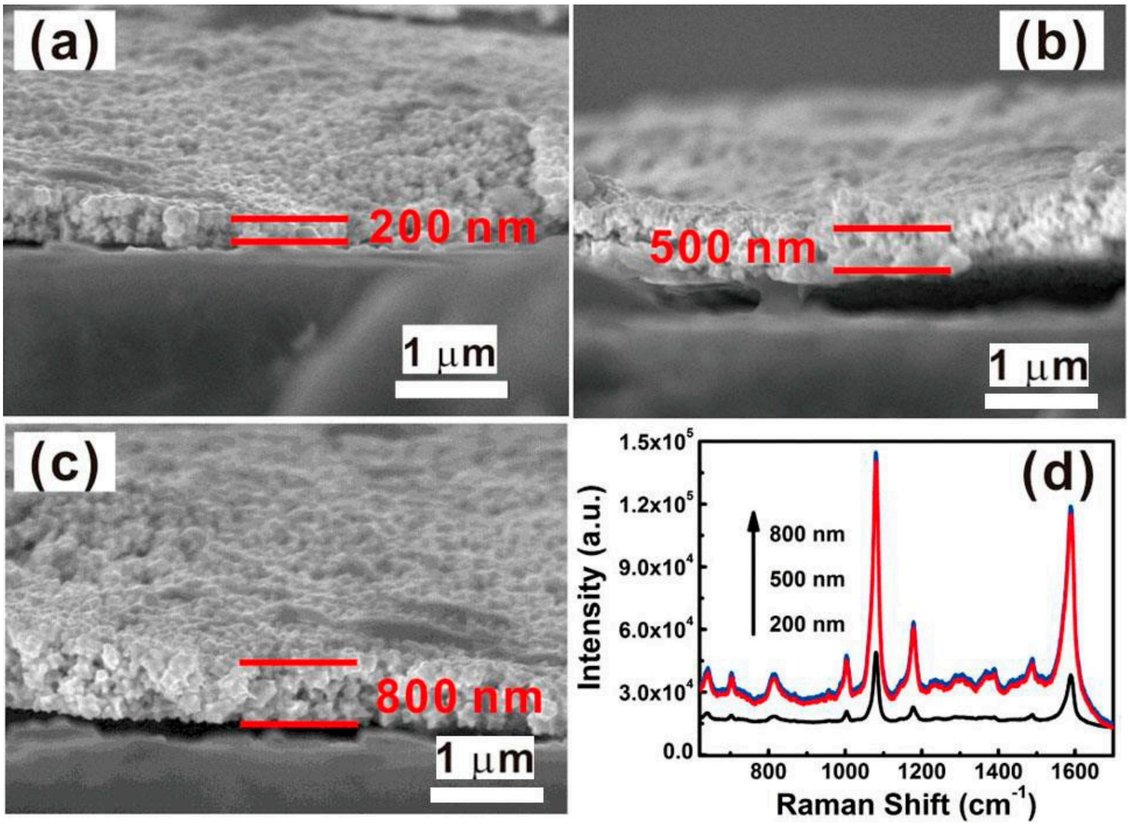

Figure S12. The FESEM images of the chestnut-like Au nanocrystals-built films with (a) 200 nm, (b) 500 nm and (c) 800 nm in thickness. (d) The Raman spectra of the films with different thickness after soaking in the 4-ATP solution (10^{-5} M) and drying (excited at 785 nm).

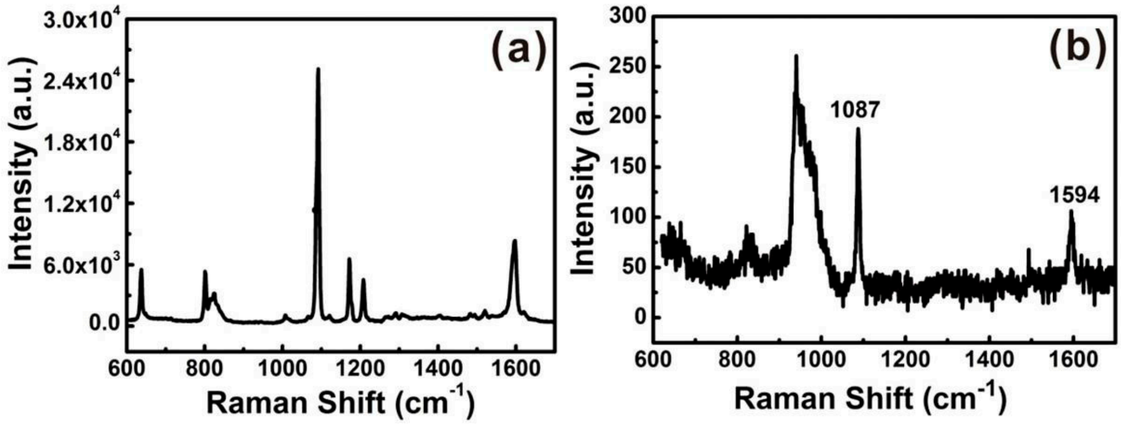

Figure S13. (a) The Raman spectrum of the pure solid 4-ATP. (b) The Raman spectrum measured after dropping 50 μL 4-ATP solution (0.1M) on Si wafer and drying. The peak around 930-950 cm^{-1} is from Si.
